# Covid-19 vaccine roll-out in England: A qualitative evaluation

**DOI:** 10.1371/journal.pone.0286529

**Published:** 2023-06-02

**Authors:** Sandra Mounier-Jack, Pauline Paterson, Sadie Bell, Louise Letley, Ben Kasstan, Tracey Chantler

**Affiliations:** 1 Department of Global Health and Development, Faculty of Public Health and Policy, London School of Hygiene & Tropical Medicine, London, United Kingdom; 2 Department of Infectious Disease Epidemiology, Faculty of Epidemiology and Population Health, London School of Hygiene & Tropical Medicine, London, United Kingdom; 3 Immunisation and Vaccine Preventable Disease Division, UK Health Security Agency, London, United Kingdom; Ateneo de Manila University Ateneo School of Medicine and Public Health, PHILIPPINES

## Abstract

**Background:**

The UK was the first country to launch a national pandemic COVID-19 vaccination programme, which was implemented swiftly despite significant vaccine supply constraints. The delivery strategy used a combination of mass vaccination sites operated by NHS secondary care providers and local sites led by Primary Care Networks, and local pharmacies. Despite nation-wide rollout, persistent gaps in coverage continued to affect particular populations, including ethnic minority and marginalised social groups.

**Aim:**

The study examined sub-national immunisation commissioners and providers’ perspectives on how the COVID-19 vaccine programme was operationalised, and how delivery strategies impacted inequalities in access to vaccination services and uptake. The study aimed to inform national programme implementation, sustainability and future pandemic preparedness.

**Methods:**

Qualitative research was conducted in eight local NHS areas in 4 regions of England. Semi-structured interviews were performed with 82 sub-national NHS and public health vaccine providers and commissioners.

**Results:**

England’s COVID-19 vaccination programme was described as top down, centralised and highly political. The programme gradually morphed from a predominantly mass vaccination strategy into more locally driven and tailored approaches able to respond more effectively to inequalities in uptake. Over time more flexibility was introduced, as providers adapted services by “working around” the national systems for vaccine supply and appointment booking. The constant change faced by providers and commissioners was mitigated by high staff motivation and resilience, local collaboration and pragmatism. Opportunities for efficient implementation were missed because priority was given to achieving national performance targets at the expense of a more flexible sub-national tailored delivery.

**Conclusion:**

Pandemic vaccination delivery models need to be adapted for underserved and hesitant groups, working in collaboration with local actors. Learnings from the initial COVID-19 vaccine roll-out in England and elsewhere is important to inform future pandemic responses, in tailoring strategies to local communities, and improve large-scale vaccination programmes.

## Introduction

England and Wales had one of the highest excess mortality rates in Western Europe by the time the COVID-19 vaccination programme was rolled-out on 8 December 2020 [1]. The UK was the first country to launch its national COVID-19 vaccination programme in response to the COVID-19 pandemic. The overall aims of the UK vaccination programme evolved over time, shifting from reducing mortality and alleviating pressure on both the NHS and the wider social care system (from December 2020) [2] to reducing disease morbidity and mortality by vaccinating lower risk population groups (from April 2021 onwards) [3].

The UK vaccine prioritisation strategy was steered by the advice of the Joint Committee for Vaccination and Immunisation (JCVI), which recommended to first immunise health and social care workers (HSCWs) and the over-80s, followed by 9 key priority groups covering all adults aged 50 years and over, and younger adults with underlying health conditions that put them at specific risk from COVID-19 [3, 4]. The Pfizer vaccine was initially provided to priority groups 1 and 2 (HSCWs and over-80s), after which Astra Zeneca was used primarily to vaccinate subsequent priority groups until the emergence of side effects linked to its ChAdOx1 vaccination led to the use of Pfizer for under 30 year olds in May 2021 [5].

Ahead of the COVID-19 vaccine introduction, public intention to vaccinate was high, though survey data was already pointing to higher hesitancy in specific ethnic minorities [6]. This evidence led the UK government to encourage local public health actors to develop a vaccination strategy that helped ensure high uptake in ethnic minority groups [7]. The rollout progressed quickly through successive priority groups [8, 9] but persistent gaps in COVID-19 vaccine coverage continued to affect a number of ethnic minority groups. The National Audit Office 2022 Report on the rollout of the COVID-19 vaccination programme in England highlighted that while all adults were eligible for two doses of the vaccine by the end of October 2021, rates were lower in more socially-economically deprived groups (75% for the most deprived decile of deprivation versus 94% for the least deprived) and in specific minority ethnic groups (48% for those or Chinese origin, 49% for those of Black Caribbean origin and Black Other origin versus 86% for the white British group 76%) [10].

As in other countries, the COVID-19 vaccination programme was implemented rapidly and affected by multiple organisational and political challenges. These included on-going changes to the programme due to vaccine supply constraints, the need to swiftly deploy different types of vaccination centres and rapidly train large numbers of staff, as well as the necessity to communicate changing evidence and policy to HSCWs, the media and the wider public [11]. The pace of implementation was described in national policy documents as “*equivalent to establishing a national supermarket business in less than a month*” [12, 13]. In January 2021, the Government translated the national strategy into an operational vaccines delivery plan that outlined organisational and delivery strategies spelt out along the areas of supply; prioritisation; places; people; and tracking progress [14].

Before its launch, there was little evidence on how to develop and implement such a large vaccination programme at pace during a national crisis. Though a country-wide vaccination programme was introduced in 2009–10 A/H1N1 pandemic, lessons learned were scant because H1N1 vaccination was rapidly discontinued in view of the mild nature of the disease and low population level uptake [15]. Nevertheless, available evidence highlighted the strategic role of local primary care and General Practice (GP) in supporting population trust in a new pandemic vaccine, and in reducing inequalities of uptake [16–18]. Evidence on the acceptability, effectiveness and efficiency of COVID-19 vaccination strategies is emerging across countries but it remains partial and fragmented [19–24]. Drawing on a qualitative study design, we examine how the national COVID-19 vaccination programme was operationalised from the perspective of providers and commissioners and the extent to which delivery strategies impacted inequalities in access to vaccination services and uptake. Our aim was to inform responsiveness to equity in the current vaccination programme implementation and sustainability, and future pandemic vaccination strategy.

## Methods

### Site and interviewee selection and consenting procedures

The study focused on regional and local organisations involved in the delivery of the COVID-19 vaccination programme. We identified 4 NHS regions that comprised of a range of geographies and socio-demographic and characteristics, such as urban/rural areas, and within each we selected several local NHS Clinical Commissioning Groups (CCG)/Local Authorities (LA). As the aim of this paper was to examine whether COVID-19 vaccine delivery models’ were able to reduce anticipated inequalities of uptake, we sampled Local Authorities to include areas of moderate to high deprivation some of which exhibit a multi-ethnic population make-up (Table 1).

**Table 1:** Interviews by staff type and affiliation.

Region	Providers	Commissioners	Local Authorities	Other	Total	Description of study sites
1. North of England	2	4	5	2	13	Urban deprived; semi-rural
2. East of England	4	5	3		12	Mostly rural
3. South of England	12	11	3	2	28	Mostly rural and semi-urban
4. London	18	11	5		34	Urban deprived
**Total Interviewees**	**36**	**31**	**16**	**4**	**87**	
*Site visits*	*5*				*5*	*2 mass sites*, *1 pharmacy and 2 pop ups*

Interviewees’ affiliations explainer: R = Region 1–4; MASSVAC = Mass Vaccination Centre; CCG = NHS Clinical Commissioning Group; LA = Local Authority; POP = Pop-up Clinic; PHARM = Pharmacy; COM = Commissioning staff

We interviewed immunisation commissioners at regional and local level, local public health professionals and vaccination providers staff in each of the region and local areas. Respondents were identified through snowball sampling and with the support of UK Health Security Agency (UKHSA then known as Public Health England) and the local National Institute of Health and Social Care Research (NIHR) Clinical research network in three regions. The number of interviewees and their organisational affiliations is described in Table 1. After staff provided informed consent, they were interviewed primarily remotely (Zoom/Microsoft Teams) and in some cases face to face. The study information sheet and consent form were sent to participants by email and returned completed by email. Verbal consent was also recorded at the start of the interview. Site visits were conducted at five COVID-19 vaccination clinics, and notes were made on the site organisation, accessibility and activities. Interviewees were asked about their experience of the delivery of the vaccination programme, characteristics of the organisational delivery models used, and how these were effective in engaging, outreaching to, and vaccinating underserved groups, the nature of collaboration between actors involved in the local area and what facilitated or created challenges to implementation. All interviews were audio recorded.

Ethical approval was granted by the London School of Hygiene & Tropical Medicine Observational Research Ethics Committee (Refs 22655), the Health Research Authority (Project ID: 20/HRA/5615), and from Research and Development departments in the study sites.

### Analysis

Interviews were transcribed verbatim and analysed using a thematic framework in the qualitative analysis software NVivo (version 11, QSR International Pty Ltd., Melbourne, Australia). The qualitative framework was developed iteratively by the team of 4 researchers using the stages outlined by Braun and Clarke [25]. We used both an inductive and deductive approach. Our initial framework to the research was informed by a rapid literature review on vaccine roll outs in previous pandemic and researchers work on vaccine preventable outbreak response. Key themes emerging from the literature review included the strategic role of local-level healthcare professionals, notably primary care teams, in supporting public trust in the A/H1N1 pandemic vaccine [16–18]. However, we allowed new themes to emerge beyond the initial theoretical framework because of our focus on how immunisation commissioners feel able to respond to inequalities surrounding vaccination services and uptake. A research team of 4 discussed and refined the coding framework after it was piloted by all researchers. Regular meetings were conducted to refine the coding framework once coding had started. We reviewed and discussed themes and developed a reporting thematic frame that would best reflect the key findings across types of respondents, organisations and regions.

## Results

A total of 87 respondents were interviewed and 5 vaccination clinics were observed between 1 February and 19 October 2021. Seven thematic topic areas emerged from our analysis of the national Covid-19 vaccine roll out and its ability to address inequalities of service uptake:: command and control; vaccine delivery models; gradual shift towards decentralisation and tailored strategy; use of data to increase uptake; managing change; value of collaboration; and efficiency.

### Command and control

The vaccination programme was led by the central government, with NHS England and the Department of Health and Social Care in the driving seat. The programme aimed to vaccinate the maximum number of people in a minimum period of time. The term “Command and Control” was used to describe the vaccination programme by many respondents. This involved the “centre” organising the rapid deployment of the vaccination infrastructure, setting up clinical and reporting systems and supply management. The political nature of the vaccination programme was highlighted by interviewees at all levels of the system:

“*There’s a political desire to make nice public announcements without thinking about the operational requirements*, *and then certainly—the central control of this government over the COVID-19 vaccination has been phenomenal*

*#R1_4_GP*.

Public Health England, the technical scientific agency at the time, and local public health and sub-national commissioning NHS organisations were reported to have been side-lined in decision making and implementation of the vaccine rollout programme, particularly at its start.

The highly centralised strategy was dominated by the NHS, with input by military planners. Interviewees in all regions noted that the programme had not articulated strategies to address inequalities of vaccine uptake. However, data quickly emerged that although vaccination uptake was high, “*[sites] weren’t getting the right people in in vaccination numbers” #R4_33_CCG* with specific groups exhibiting low uptake—notably Asian and Black ethnic minorities. Performance management of the programme was stringent, involving “*a huge amount of scrutiny up and down” #R3_60_COM* and included regular review of vaccination numbers and cohort penetration, accompanied by corrective actions required of providers. The minimum target of 85% coverage in each cohort meant that lower performance became especially visible in less homogenous communities, such as multi-ethnic urban areas. Even in a predominantly lower density area, *“rurality and access didn’t seem to be the biggest issues*, *it definitely seems to be age and deprivation”* that seem to explain poorer uptake #*R3_64_COM*. One interviewee noted that

“*Command and control might have had a plus on one level*, *but it was a disaster to an area like ours*, *which needed a tailor-made approach*”

*#R4_59_CCG*,

while in another region it was observed that

*“It’s fair enough having a national plan*, *but I think it should’ve been devolved more for flexibility on the ground*, *on a local basis*”

#*R3_76_GP*.

The consensus of those with public health experience was that community engagement and the use of tailored delivery models was needed to ensure high uptake in these settings. But as one interviewee put it, the change of direction could be slow, observing that it was

“*Really difficult for the NHS and the metaphor of course is the oil tanker slow to start and gets go [going] but if you want to turn it round it takes forever*”

#R4_38_COM.

There was no centralised fund for these areas to catch-up although additional funding was made available later.

Centralised control was portrayed as a “Push” (vaccines being pushed from central level) rather than a “Pull” (vaccine orders placed by sub-national levels) strategy for managing vaccine supply, which contributed to the lack of agency and control reported by providers. Vaccination providers recognised genuine supply challenges and some praised the work of the national supply management team. However, providers lack of control was described as an impediment to the effective management of bookings and staff resources:

“*I don’t know*, *both quantities or the type of vaccine*. *So*, *some weeks you get Pfizer other weeks you get Astra and you had no knowledge*, *which was coming until right up until the last minute*. *And that’s hugely problematic when you’re trying to organise staff rotas and everything*”

*R4_47_GP*.

Some bottlenecks were viewed as inevitable, but others were regarded as deriving from rigid management systems.

Lack of control was repeatedly reported as a major frustration by providers—control over supply, bookings and booking systems, clinical protocols, priority groups eligibility—all of which had implications for managing vaccine delivery, scheduling staff, and overall efficiency. National protocols did not authorise vaccine dispersal to multiple sites such as individual GP practices and community sites. This made access more difficult for community members which were more hesitant to travel to unfamiliar settings. Some of these constraints were the results of manufacturers’ cold chain and transport requirements (e.g. Pfizer vaccine being initially challenging to transport and store) but providers perception was that these were applied rigidly, even when dispersal was technically possible (with Astra Zeneca vaccine which did not face transport and storage challenges). In several cases, GPs reported having to drive every morning to pick up 10 vials of the vaccine rather than being able to place a week’s provision in their fridge:

“*And he’ll do that six days a week*, *which is a huge waste of time in terms of his time*, *but also from our point of view I have to have someone on site to be able to give him*, *six*, *seven*, *bottles of vaccine every day*, *which is just bonkers*, *absolutely bonkers*. *Whereas if I was allowed to give him his weekly supply*. *And then he could stick in his fridge and just use it through the week*. *That would make much better sense but we’re not allowed to because of the vials under the licence*”

*#R4_47_GP*.

Another example was the challenge to share vaccines across sites in a given locality with any transfer of vaccines between sites requiring lengthy procedures and multiple signatures for authorisation. Nevertheless, one local area reported a functional system of mutual aid that was facilitated by relationships and trust: *“So if one site hasn’t got any*, *we can generally find another site who can lend them some”* #*R2_24_CCG*. Another example was the inability of providers to vaccinate members of the same households who had been booked on different dates, requiring people to come back or otherwise staff having to complete a large amount of paperwork:

*“People will be coming*, *so the elderly would come through*, *and they would come—you know*, *the spouses would come together*, *and husband had his date for that date*, *but the wife was supposedly going to come back the next day… And*, *you know*, *you can’t send these people away and say”*, *“No*, *you’ve got to come back tomorrow*”

#R1_4_GP.

However, one Primary Care Network (PCN) provider acknowledged they did vaccinate someone turning up with someone who had been booked in, and that they did so “*despite the system*” #R4_59_CCG.

Communication was also centralised and described as politically driven: “*it felt like there was the national way that this had to roll out*, *and locally everybody just had to fall in line*” #R3_70_CCG. In some instances, this undermined locally-developed communication strategies:

*“We’d gone a long way down a path of designing a local communications thing and then that was*, *‘No*, *you can’t do that because it’s*, *we’ve got a national thing coming out*.*’ But you know*, *you’d worked*, *co-produced some of the materials and things like this*. *So*, *but yeah*, *there’s been a lot of that where good intentions and what’s possible aren’t necessarily realised*.”

#*R1_13_CCG*

There were some tensions in cohort prioritisation but also a recognition that rules were bound by the JCVI guidelines. In some deprived areas, respondents argued that age could be a blunt criterion where younger people tended to be in poorer health. It was felt that the JCVI strategy could increase local inequalities of access:

*“There was no recognition that we should be able to open to younger population*, *because—just to put it very starkly*, *in the city our population don’t live into their 80’s*..*so when you look at it on paper*, *it looked like we were prioritising the more affluent*”

#R1_3_CCG.

Others considered that later on, this approach prevented younger groups of ethnic minorities to act as ambassadors for their possibly hesitant elders. Respondents in two deprived localities suggested that vaccinating all members of multigenerational households would have been a good approach. Likewise, classification using narrow population groups was found to be unhelpful, as well as, for example with the homeless being considered in the prioritisation ahead of asylum seekers who some interviewees felt had very similar health needs. However, one interviewee argued the risk was for “*everyone*, *you know*, *(to) create your own mini JCVI*,*”* #*R1_12_Com*. The cohort prioritisation had unintended consequences for more hesitant groups, or those with limited access to services, who were dropped from prioritisation by local providers who concentrated on the next cohort:

“*We had higher vaccination rates in our earlier cohorts because*, *again*, *we were forced to kind of stay within that boundary*. *Whereas London*, *now they—you know*, *everyone’s blinkers are still on for the low-hanging fruit*. *So as soon as you move on to another cohort*, *you kind of stop thinking about this because you’re passing*, *you’re moving at scale for these next age groups*”

*#R1_12_Com*.

However, by the time cohort 6 were eligible for vaccination, local providers were reporting a more flexible interpretation of the JCVI guidelines. In one locality, homeless people were vaccinated ahead of authorisation, with the justification that “*you go and do what’s best for our patients and risk assess and make decisions”* #R1_1_GP and in another the PCN reduced the interval between dose 1 and 2 to 28 days to ensure vaccine completion among homeless people.

### Vaccination delivery models

Initial plans established before October 2020 suggested that mass vaccination centres would be delivering the bulk of vaccinations, complemented by small community clinics and roving vaccination models. However, from October 2020, in the run-up to the COVID-19 vaccine introduction, local vaccination services (LVS) such as GPs and pharmacies were added as new delivery “pillars.” The actual roll-out involved delivering phased tiers of vaccination provision, starting with a) hospitals and national mass vaccination sites which vaccinated HSCWs, b) primary care networks (PCNs) which enabled GPs to organise vaccine delivery in a limited number of larger sites, and then c) pharmacies and community clinics (such as pop up clinics delivered in community centres, or religious settings) were added (Table 2 for additional details). One reason why primary care had not been originally included as a delivery pillar was reported to be that the NHS *“wanted primary care focused on kind of like business as usual” #R4_28_COM*

**Table 2:** Key vaccination delivery model typology.

Models	Description and characteristics
Mass vaccination hub/site	Contracted by NHS England through block contract based on estimated target population Commissioned nationally or regionally by NHSE. Location based on available physical appropriate site, and criteria of access (car/public transport).
Primary care: Primary Care Network (PCN) and GP delivery models	Funded through Fee for Service (£12.83 per vaccination). A PCN is a collaboration of GP practices pooling resources and patients list to provide vaccination services. PCNs usually deliver vaccination through a few large sites within a local NHS clinical commissioning group (CCG) footprint. May be based on existing GPs grouping (eg. GP federation) or one that is newly developed.
Pharmacies	Funded through Fee for Service (£12.83 per vaccination) Commissioned by regional NHS based on site suitability and location, once assurance process completed.
Pop up clinics	Funding based on each providers contract. Delivered either by PCN through smaller vaccination sites (dispersal) or through mass vaccination centres led by Acute NHS Trusts. Location sites selected based on community needs and site suitability (Faith-based such as Mosques, community centres, outreach sites for transient population, vaccine buses…etc).
Other	Hospital based vaccination within hospital wards and targeted at health and social care workers and inpatients. Housebound outreach services: GP led or provided through Community Trust services Care home vaccination: GP led or provided through Community Trust services Integrated vaccination services (providing holistic preventive and some curative services to a specific population).

The vast majority of interviewees observed that the range of vaccination service offers was pivotal for addressing the diverse needs of people and settings. However, GP-led PCN sites reportedly took a central role in the roll-out. One interviewee observed that while mass vaccination hubs operated by large hospital Trusts had been expected to deliver the bulk of the vaccines, primary care had ended up delivering the vast majority (over 75%) by the end of 2021. This was noted as “*unsurprising”* by interviewees, given GPs’ track-record of conducting seasonal flu vaccination; their well-trained staff; being physically closer to communities than mass sites; and trusted relationships with patients, “*which is so important*, *particularly for a new vaccine” #R4_32_GP*. As one interviewee put it, *“It’s just nice for those who really struggle just to get that little bit further*, *it was nice to have—offer them the familiarity as well because the surgery” # R3_75_GP*.

The location of mass vaccination sites was mostly decided centrally and public health authorities and NHS commissioners/providers had reportedly limited input in the location’s decision. There were practical reasons for the choice of locations, including space availability, requirement for social distancing and transport links. However, the lack of local consultation was criticised by sub-national actors: “*I think the NHS were aware of the sites but were sworn they couldn’t tell anybody though invitation letters were already going out”* #R3_62_LA. Interviewees, including those of mass sites reported that mass vaccination sites were under-utilised as noted by one interviewee

“*Some of the mass vaccine centres were expecting this massive amount of throughput*, *which just hasn’t materialised*”

# R3_85_MASSVAC.

Underlying factors of poor utilisation included being located too far from local populations; difficulty of access for those without a car; concern by those shielding about using public transport; lack of familiarity and reluctance to leave local communities; and in some case multiple mass sites being situated within a few hundred meters of each other. In contrast, PCN site locations were decided in consultation with local GPs. However, the number of permitted PCN sites was limited, leading to large geographical catchment areas—such as only one site allowed in a large Northern city.

*“At the beginning there was absolutely no freedom whatsoever with regards to how we did it*, *because otherwise we would have opened the site in the city centre in the original wave*…*Yeah so I think we would have liked to have been more flexible in our deprived population*”

#R1_3_CCG.

It was hypothesized by one interviewee that the limitation in the number of vaccination sites was designed to manage supply constraints.

“*They wouldn’t let people have more than one site*. *…From the outset*, *we’ve always said*, *“If you give the vaccines to GPs and let them do it the way they normally do vaccines and the supplies were OK we’d have been finished by now*”

#R1_1_GP.

However, gradually over time, more sites were allowed to open in successive waves and those were located closer to where local residents lived.

Providers at local level reported delivery “pillars” being somewhat siloed at local level, and some pointed out that this led to a lack of understanding of who was doing what: “*No*, *we’re not linked with anybody really*. *I mean from a system point of view you’re aware of who’s doing what but no real direct communication”* #*R3_85_MASSVAC*.

Mass vaccination centre leads stated that they had no knowledge of local PCN providers and there was no mechanism for pillars to communicate with each other, though at commissioning levels there was some coordination between pillars. Sometimes, there was overt competition between pillars fighting for the same patients noting that these were “*fishing in the same pond [of patients]*” as different providers aimed at achieving their performance targets. This led one interviewee to note that better provider delivery integration at local level would *“really would have made a difference” #R4_28_COM*.

### Gradual shift towards decentralisation and tailored delivery strategy

Confronted with the need to achieve high uptake in underserved minority ethnic groups, there was a gradual re-grouping of local systems and an allowance for greater flexibility in the delivery of the programme. As one interviewee put it:

“*And this was very sort of top down*, *quite*, *quite fragmented way of delivering …and we had to start to coordinate between us*, *a lot better because we had to defragment some of that way in which we sort of established and commissioned*”

#R4_35_CCG.

Yet, interviewees lamented that the shift towards more flexibility had been slow to materialise and as described by a regional commissioner: “*So I think it’s fair enough having a national plan*, *but I think it should’ve been devolved more for flexibility on the ground*, *on a local basis”* #R3_76_GP.

One interviewee commented that the shift to more local flexibility stemmed from the national level *“losing interest in the programme*” #R4_59_CCG as with its performance targets achieved and society reopening from restrictions, its political prominence reduced. Most interviewees reported that by the end of spring 2021 they had become “*much more flexible and differentiated in [our] approach*” R4_48_MASSVAC and that “*Director[s] of public health [were] being able to set local priorities somewhat outside of the [JCVI] age cohorts”* #R1_12_Com. This new autonomy soon translated into local approaches for reaching under-vaccinated communities and the implementation of innovations, such as with pop up clinics in urban centres and workplaces. Thus, the programme morphed from “*delivering as many vaccinations as possible [to] ensuring that we also deliver it equitably*” #R4_33_CCG, and tailored models opened *“on the doorstep [of local communities] to make it accessible to them*” #R4_43_POP.

When decision-making about site locations was shifted to local areas, some vaccination providers decided to implement vaccine dispersal to smaller general practice led sites, open pop-up clinics and in some cases reconfigure their mass vaccination sites, including closing some and opening new ones to better fit local needs. This also involved expanding the number of pharmacies, and tailoring delivery to younger cohorts by using popular venues such as shopping centres, football matches/sporting events and festivals to address the needs of a population target group that had traditionally less contact with GP practices.

### Use of data to increase uptake

Understanding reasons for lower uptake was seen as critical to improve programme performance, identify areas of lower uptake and reasons for it, and support the design tailored vaccination delivery models:

“*There will be different things that you need to do for different people*. *So*, *it can’t just be a numbers game*. *So*, *there’ll be specific reasons why one area might be at 90%*, *and then another might be at sort of 68%*”

*#R4_37_CCG*.

There was a range of factors cited for under-performance such as areas having a smaller demographic of over 80-year-olds; a larger demographic of younger people; and migration of young people in recent years not matching the census population stratification. Some of these factors were complex and often rooted in local health inequalities—which according to local actors were often not well understood by national stakeholders

“*People in power*, *think*, *well*, *what’s the problem*? *Why can’t people just get in their car and drive to [a mass vaccination centre] and get their vaccination*? *Because that’s how their life [is] […] they don’t understand that [this area] is a completely different world*-”

#*R4_59_CCG*.

One public health stakeholder commented that *“we’re going to get there*, *we’re just gonna get there more slowly*. *Just an expression of hesitancy” #*R4_41_LA, noting similar poor performance in other screening programmes and the need for enhanced engagement.

Local areas set up “intelligence hubs” where vaccine uptake data was analysed to inform the design of outreach strategies: “*You know*, *do the analysis or the modelling and we test out different ways of reaching people*, *and that sort of informed a lot of our outreach work*” #R4_36_CCG. However, one respondent noted that modelling “*had not been that useful”*, as those involved had not been familiar with local health data. Local authority public health departments also conducted extensive population polls and focus group discussions, to explore reasons for non-vaccination and develop engagement strategies with under-vaccinated groups.

### Managing change

The vaccination programme was described as *a “roller coaster and never knowing what the next day will bring*, *while trying your best to muddle through”* #R4_50_MASSVAC, which led to some respondents commenting that they operated in a “*reactive mode*” #R4_56_PHARM. Changes came regularly, whether supply shortage issues, change of vaccine(s) expected or not and with variable cold chain needs, opening of new patient cohorts with different characteristics, change of JCVI policy recommendations such as increasing the interval between doses 1 and 2, and following adverse events for Astra Zeneca vaccine, and shifting to younger cohorts at the same time some GPs and PCNs were disengaging from the programme.

The constant flux of changes led some people to speak about their “*resilience being tested*” #R2_20_COM: and having to “*build this programme while we were flying it”* #R3_61_MASSVAC. Learning was reported to be continuously shaping and reshaping the programme. Many respondents highlighted a high level of stress and requirements to provide a large amount of information up the system under extreme time pressure, and out of hours: as one interviewee noted

“*The amount of hoops we had to jump through I can’t even describe the number of emails we would have at 10 o’clock in the morning*, *you need to reply with this information by 2 o’clock today*”

#R3_81_GP.

Communication within the vaccination programme was criticised as being non-transparent and last-minute. It made adapting to change complicated:

*“We were sitting there at 10 o’clock watching the news waiting to see what he [PM]’d say*, *because then we’d have to spend the next of couple hours sometimes planning for the next day*, *to deal with the aftermath*”

*#R2_20_COM*.

One consequence of the untimely dissemination of clinical information noted by one provider was that “*patients that turn up to the service lose confidence in it*, *because the person vaccinating you doesn’t “know” as much as you do”* #R4_54_MASSVAC. Despite some recognising the huge challenge faced by the central national vaccine team which was described as *“very sharp*” by one interviewee, many lamented a lack of feedback loops from the centre to address challenges. There was a general perception among local actors of a lack of recognition of the complexity of the task in areas of low uptake:

“*There is a sort of sense that programmes are what they are*, *I think there’s a sense of this is the big success story*. *And the fact that we have been working flat out since late November*, *to tailor and customise the programme […*.*] perhaps has not landed*.”

*#R4_41_LA*.

The lack of a feedback loop was illustrated by the inability of mass vaccination sites and pharmacies to reschedule appointments and one mass vaccination site reporting having *“escalated it [the issue] on a number of occasions up through system and regions have escalated to national” #R4_49_MASSVAC*. Outreach was described as challenging to organise because of the lack of dispersal policy to additional sites and challenges in accessing funding and having to go through a bidding process, leading to delays in reaching an often-vulnerable population. Some noted that bureaucracy was unavoidable as this was in the blood of the organisation: “*NHS was never designed to be a personal health service*. *So*, *trying to do things in microcosm is very difficult for us” #R4_38_COM*. Broader shortfalls were also attributed to lack of flexibility in the forthcoming NHS organisational restructuring and anxious middle managers’ rigid adherence to guidelines. #R1_1_GP.

Nevertheless, more pragmatic approaches were adopted as the programme evolved: “*it’s better now*, *it’s a bit more flexible now*, *but then it was very*, *very regimented […] we have just tried things and if it hasn’t worked*, *we’ve reflected on it*, *evaluated*, *and moved on*. *And so*, *it has been completely pragmatic*” #R2_21_AT. This pragmatism also involved a shift in the architecture of provision, with some PCNs withdrawing from the programme and pharmacies and local community centres being brought in.

Interviewees were often positive about how the impetus to adapt continuously to changes had enabled them to work differently: *“From an NHS perspective those conversations would normally take about five years and we had to get it done and built in five weeks” #R3_85_MASSVAC*. It had also incentivised stakeholders to work more holistically in tailoring services to needs. Working with communities and engaging communities was seen by many as the real expertise required. It was portrayed as adopting a kind of “*action learning approach*”, consistently listening to conversations within the community and incorporating patients’ feedback into programmatic changes and new tailored delivery models:

“*It’s about valuing those people in those communities who can’t get access for the vaccination*, *for whatever reason*, *and providing them the ease and convenience of getting it can make such a difference*”

*#R2_25_LA*.

Navigating changes and achieving programme performance relied on intrinsic staff motivation and staff having a “*common goal with […] everybody’s singing from the same hymn sheet” R3_67_CCG*. Team cohesiveness was described as crucial and many referred to a “*war spirit*.” Clinical staff also often remarked that they saw their involvement in the vaccination effort as a positive way to leave the painful experience of the pandemic behind. However, many staff reported being stressed and sometimes feeling their work was not being recognised, notably in primary care, commenting that they *“were on a treadmill*, *and there’s no off button”* but recognising “*it [would not be] feasible for us to stop right now*. *I think it would just fall apart” #R4_43_POP*.

### The value of collaboration

An important feature of the vaccine rollout programme was that most players involved in commissioning and delivering immunisation were not those who had these roles or experience in the past. NHS acute hospitals, acting as leads for the NHS Integrating Care Systems (ICSs), led the vaccine programme, though they were not traditional actors in the immunisation programme, a role that was previously devolved to CCGs. Furthermore, acute Trusts were reportedly not accustomed to local collaborative working in the realm of public health. Tensions were described between the “acute” Trust leadership and other local NHS actors who felt the former *“were biased against public health prevention” #R4_59_CCG*, and did not understand local immunisation contexts while regularly blaming poorly performing areas. In one region, the local committee, which was dominated by acute providers, was focused on wider system structural change at the expense of the operational steering of the vaccination programme. This might be a reason why directors of public health were not involved in the planning and implementation activities of the programme, which resulted in frustration at local level. More generally there was poor communication with local actors—especially at the start of the roll out, which impeded engagement efforts at community level.

Overall, the governance structure was quite complex, with different lines of accountability. Lead acute NHS Trusts were responsible and accountable for all mass vaccination activities, NHS-England, through its regions, delegated its commissioning responsibilities to CCGs and primary care networks, while pharmacies were contracted directly from commissioning NHS regional offices.

Actors started to work more collaboratively, with workstream leads meeting frequently, though collaboration remained initially confined to NHS staff. Providers groups were also set up to help coordinate vaccine delivery. Gradually the system regrouped organically at local level and CCG-led coordination fora emerged, and were used by providers to solve problems. There was a recognition that communication within the system had improved as the programme matured, also facilitated by digital technology.

Public health stakeholders—which had a peripheral role in the roll-out initially—became more engaged in the vaccination programme though this was often helped by historical links between NHS and public health and personal relationships. Public health staff often took a leading role in advising on and leading work on inequality of uptake. Collaboration was praised: “*Ultimately*, *we got over that hump quite quickly and we set up a really good partnership arrangement but we had the right people around the table which was the success factor”* #R3_62_LA. One interviewee argued that the NHS had little choice than to collaborate with local public health officials to address local needs as it had had “*zero ability to control communications*, *to do some kind of innovative campaign that targets certain groups*, *unless [they] kind of do it through a local authority”* #*R1_12_COM*, giving a particular incentive to working collaboratively. NHS respondents commented that despite its limitations, collaboration with public health authorities was much improved compared to the Test and Trace programme which had been outsourced to private providers:

“*We are definitely*, *by this point again*, *fairly well joined up I think part of it is that the Director of public health meet weekly*, *and the heads about boroughs attend that meeting*, *so they can be updated*, *they can feedback*”

*#R4_33_CCG*.

An important step was some regions and local areas set up vaccination multi-stakeholder inequalities groups to address the needs of under-vaccinated communities. One interviewee noted the complementarity between the NHS’ strong programme management skills and LAs’ strategic approach in delivering the programme. However, this was not helped by early data sharing issues: “*I think*, *you know*, *initially*, *we weren’t even allowed to share the numbers of vaccinations that happened in each borough with local boroughs*” #R4_33_CCG. However, on the ground, providers such as GPs reported continuous trusted collaboration with public health staff in local authorities.

Collaboration was described as pivotal to programme performance: *“*

*The overall learning is that the best impact happens when the NHS and local authorities work closely together*, *because of the different knowledge*, *that those two different bodies bring about those communities*, *and also because of the kind of the different relationships that we have with those communities*”

*#R4_42_COM*.

This was particularly critical in large multi-ethnic urban centres where granularity of knowledge underpinned engagement and where local authorities’ connections to communities and local voluntary organisations could be leveraged.

A positive legacy of this collaboration was the resulting strengthening of newly formed regional commissioning organisation like Integrated Care Systems, and better interpersonal relationships as explained by a regional commissioner: *“I know 20 of the 33 local authority chief execs by first name now*. *I didn’t do any of them before*. *They know me*. *Is that a good thing*? *I think is a good thing*” #R4_30_COM.

### Low emphasis on efficiency in the national strategy

Interviewees recognised that the national programme had been extremely well funded but many noted that efficiency did not really feature in the strategic decision-making, with emphasis being placed on programme performance and achieving high uptake. Inefficiencies were rooted in the siloed providers funding model which led to duplication of capacity and did not encourage collaboration. Under-utilised and costly mass vaccination centres which were funded by block contracts were reported as inefficient. Various clinical vaccination protocols were implemented in vaccination centres and providers had contrasting views about their efficiency. For instance, the *Patient Group Directions* (PGDs) which provide a legal framework that allows some registered health professionals to supply and/or administer specified medicines were assessed by some interviewees as costly to operate. Multiple data recording systems were described as poorly integrated and inefficient, though interoperability seemed to have improved over time. The lack of flexibility of the national booking systems resulted in providers not being able to reschedule appointments, which in turn led to unused capacity and unattended appointments.

*“If inter-operational booking systems allowed centres to have their rescheduling functionality delegated to them*. *I do think you know we’re responsible NHS providers*. *And I just do think that that is adding a huge amount of inefficiency into the system*.”

#*R4_49_MASSVAC*

Call/and recall systems were criticised as duplicative, with multiple invitations being sent by providers, and sometimes inefficient, with providers requiring a large amount of manpower to book an increasingly small number of unvaccinated patients.

Conversely, many felt that funding for outreach strategy may have been insufficient and often hinged on accessing time-limited and competitive funds. However, when available, the funding of community engagement and outreach activities was perceived as a valuable long-term systemic investment:

“*Better collaboration with local communities which can help strengthen other preventative programmes such as flu or screening*. *And so when you’re looking at it from an efficiency perspective*, *you’re thinking*, *well*, *that’s an awful lot of resource time*, *energy*, *for 20 people to be vaccinated*. *But when you look at the knock-on effects around the long-term health of those people*, *and the relationship*, *it broadens out beyond health*”

*#R3_70_CCG*.

Finally, many interviewees felt that because of lack of initial delegation to sub-national levels and the fast pace of implementation, there had been limited opportunity for evaluating delivery models and to remedy potential inefficiency.

## Discussion

The COVID-19 vaccination roll-out in England was, in many ways, an unprecedented programme. It achieved its numerically ambitious targets such as vaccinating 14.6 million people within less two months of its start [22], and going beyond its target of two doses received by 85% of people aged 18 and over in England by October 2021 [10]. By nature, it faced multiple challenges and most of all, the need to deliver vaccines quickly on a mass scale, while having to adapt to multiple programmatic changes. The combination of time-bound imperatives favoured a strong chain of command for which the NHS was well suited but also it required local adaptations to respond to gaps in coverage most notably in multi-ethnic and socio-economically deprived communities. Necessary implementation trade-offs led to tensions arising between the goal of achieving speed and performance and for developing delivery strategies tailored to the needs of these under-vaccinated communities. Tensions also manifested through the verticality of the programme, notably the centralised vaccine supply “push” management model; poorly integrated data recording and management systems; top-down communication messaging strategy; the initially centralised organisational deployment of vaccination sites; rigid booking systems; and the strict clinical and procedural guidelines. There was a recognition that a “command and control” model was well suited to managing significant logistical and organisational challenges of the roll-out in the context of constant changes in supply and vaccine recommendations (see S1 Table). However, consensus within our study of sub-national commissioners and providers was that the lack of consultation with local actors in the design of initial COVID-19 vaccine delivery and engagement strategies had also led to more inequalities of vaccine access and uptake, and decreased efficiency.

Sub-national interviewees underlined constraints related to the rigidity of centrally designed systems, lack of delegation to actors more cognisant of local needs and the political nature of the programme which reinforced centralised control of decision making, especially during the first phase of the roll-out. They also described how with enhanced collaboration at local level, the local public health systems regrouped to deliver tailored vaccination strategies that better responded to communities’ needs, as other have also noted [21]. Crucially, building on the central role taken by primary care, vaccine delivery models gradually shifted closer to communities experiencing hesitancy and low uptake, as has been described in other contexts [26–28]. Local commissioners in our study highlighted that they had to climb a steep path to be able to eventually design local delivery strategies that were able to close this gap in the areas they are responsible for. However, and despite significant investment in engaging and reaching out to these communities, there was still a residual gap in vaccination coverage in some ethnic population groups (e.g. Black and minority ethnic groups) [10].

Vaccination providers often described “*working around”* the national systems and how supporting systems gradually became more flexible which facilitated locally designed strategies to improve accessibility. Key ingredients of the success of the vaccination programme were not described as systems related, but as driven by human factors such as commitment, passion, team cohesiveness and the nurturing of local relationships and collaborations. As seen in other public health contexts, the value of collaboration between health actors, local government and communities was recognised as pivotal to promoting confidence in the COVID-19 vaccine programme, increasing uptake and reducing inequalities [21, 29]. However, despite these organic adaptations, vaccination delivery models were still considered to be poorly integrated at local level, as the emphasis on performance and providers incentives drove competition rather than supporting local integrated delivery.

Learning from the roll-out in the UK and elsewhere is important to informing the future of the COVID-19 vaccination programme and levering its legacy to improve the delivery of public health interventions. Future pandemic response require attention to coordination among the complex framework of vaccine delivery in the UK and elsewhere. Pressure points could be avoided, efficiency increased, and tensions eased by providing a feedback loop informing national policy and giving permission to local actors to adapt their delivery strategy to local needs.

### Strengths and limitations

Our study provides the views of sub-national respondents, who may have been unaware of constraints faced by national programme managers. We sampled respondents in a limited number of local areas, which is unlikely to be representative of all organisations. However, we purposely chose regions that differed in terms of urban and rural makeup and socio-economic characteristics, which included some settings with lower COVID-19 vaccination uptakes than national rates. Our recruitment was not balanced across regions, with more views from London and Southern region due to the size and organisation of commissioners and providers, and our ability to recruit. Despite this limitation, we were successful in each region in getting the views of the different actors (providers, commissioners, and local authorities) and triangulating their perspective. We were also able to compare views across types of interviewees in all regions. Because of the overall size of the sample, we feel confident in the validity of our results. The roll-out strategy changed over time, and interviews were conducted for the most part between April and July 2021 so will not generally reflect changes in the programme that occurred later, such as vaccination of children and younger people, and the provision of the third dose of the vaccine. We cannot exclude respondents’ response bias, though the number and range of interviewees in each local area and across areas allowed us to triangulate views and increase confidence in the evidence collected.

## Conclusion

Vaccination has been central to the UK government pandemic response and has operated in a complex and fast pace environment and under important logistical and organisational constraints. The UK COVID-19 vaccine programme took a highly centralised approach, which had some benefits such as consistency and clarity, and it achieved its overall vaccination targets of providing 2 doses of the vaccine to 85% of the population by July 2021. However, the programme was less able to address inequalities in uptake in some communities and ethnic minority groups. Trade-offs between a top-down and bottom up-approaches had to be made, and gradually those two approaches became more complementary. Encouraging an early feedback loop between local actors and national level, would have improved and accelerated the closing of the vaccination gap. Pandemic vaccination delivery models need to be adapted to underserved and hesitant groups, working in collaboration with local public health actors while giving more flexibility to local systems. Future pandemic responses need to balance centralisation and local autonomy if countries like the UK wants to reduce vaccination inequities, and improve efficiency.

## Supporting information

S1 Table Systems barriers reported by interviewees. (DOCX)
