# Supplementary material for: Covid-19 vaccine roll-out in England: A qualitative evaluation
Source: PLoS One. 2023 Jun 2;18(6):e0286529. doi: 10.1371/journal.pone.0286529 (PMC10237459; doi:10.1371/journal.pone.0286529)
Supplement: S1 Table — (DOCX) [file pone.0286529.s001.docx]

**Supplementary table. Systems barriers reported by interviewees**

| Systems Barriers | Description | *Examples of challenges and* mitigation strategies |
| --- | --- | --- |
| Supply | - No control by vaccination providers of supply replenishments (push system). Described as “*feast and famine*”, notably when change of vaccination cohort. Lack of information on forthcoming deliveries. - Logistical challenge to distribute and disperse Pfizer and subsequently AZ vaccines to smaller local sites due to strict protocol | *Lead to challenges in planning staff rota; to adjust number of patients invited on* the day *with possible cancelling or advancing of booking invitations*; *and in some case vaccine wastage*. Strategy: Some areas shared supply across providers.  *Providers had to fetch and return vaccines daily as they were not allowed not store in local fridges;* later rules were gradually relaxed with the introduction of new vaccine protocol, which enabled community sites to keep weekly doses in the fridge. |
| Data systems and data sharing | - Vaccination data not (initially) shared with local directors of public health or with other stakeholders. Initial concerns on data sharing and impact on data protection. - Identifier for unregistered patients (e.g homeless) unavailable so difficult to follow up with second dose. | *Local authority’s communication and engagement strategies initially not data driven to target areas and communities of low uptake.* Strategy: some local authorities had pre-existing data sharing agreements so could engage and outreach communities early.  *Homeless not receiving vaccine second dose.* Strategy: Second dose given with shorter interval or in opportunistic manner. |
| Communication | - Programme changes announced nationally before being communicated to NHS commissioners and providers. - Complexity of guidance, and lack of digestible highlight of clinical and organisational update for staff. - Generic communication not always adapted to specific community needs. | *Reported to undermine confidence of patients in clinicians; last minute change resulted in important challenges in terms of planning and staff rotas.*  *Challenges to identify relevant new information and understand changes in guidance could lead to confusion.*  Local areas gradually developed their own communication tailored to staff and the needs of communities of low uptake. |
| Invitation/Booking systems | - Fragmented booking systems across different providers. - Some providers using the NBS (massvac, pharmacies) could not reschedule bookings. - Some providers have more flexibility (eg GPs) to invite patients and started inviting new cohorts early. - Initial challenges to book for those with no digital access to appointment systems | *Consequences: Empty slots; possible loss to follow up; inefficiency (low patients’ throughput); vaccine wastage.* Some providers stated they managed “work-around” the National Booking System (NBS).  Local authorities established hotlines to support booking; some walk-in centres were set up. |
| Call and recall systems | - Range of call/recall strategies and different providers/commissioners in charge. Initially no recording of decliners and reasons for decline. | *Consequences: Time consuming calls to patients by GPs and other providers. Some patients have already been vaccinated elsewhere.* |
| Funding/incentives | - Differentiated funding models for vaccine delivery. Fees for service for GPs and pharmacists (£12.83 per vaccine), block contract for mass vaccination led by hospital Trusts. | *Different incentives lead to competitive behaviour and possible lack of cooperation between local providers; over-capacity and inefficiency for underutilised mass vaccination sites*.  Some mass centres decided to repurpose unused capacity to staff special clinics, outreach team and pop up clinics. |
